# Conservation of Neotropical migratory birds in tropical hardwood and oil palm plantations

**DOI:** 10.1371/journal.pone.0210293

**Published:** 2018-12-31

**Authors:** Ruth E. Bennett, Wendy Leuenberger, Bianca B. Bosarreyes Leja, Alejandro Sagone Cáceres, Kirsten Johnson, Jeffery Larkin

**Affiliations:** 1 Department of Natural Resources, Cornell University, Ithaca, NY, United States of America; 2 Conservation Science Program, Cornell Laboratory of Ornithology, Ithaca, NY, United States of America; 3 College of Environmental Science and Forestry, State University of New York, Syracuse, NY, United States of America; 4 Universidad de San Carlos de Guatemala, Ciudad de Guatemala, Guatemala; 5 Asociación Guatemalteca de Historia Natural, Ciudad de Guatemala, Guatemala; 6 Indiana University of Pennsylvania, Indiana, PA, United States of America; 7 American Bird Conservancy, The Plains, VA, United States of America; University of South Carolina, UNITED STATES

## Abstract

Tropical forests in the Americas are undergoing rapid conversion to commercial agriculture, and many migratory bird species that use these forests have experienced corresponding populations declines. Conservation research for migratory birds in the tropics has focused overwhelmingly on shade coffee plantations and adjacent forest, but both cover types are now in decline, creating an urgent need to evaluate conservation opportunities in other agricultural systems. Here we compare how a community of 42 Neotropical migratory bird species and a subset of five conservation-priority species differ in usage and habitat associations among a secondary forest baseline and four expanding commercial plantation systems in Guatemala: African oil palm, teak, rubber, and mixed-native hardwoods. We found that mixed-native hardwood plantations supported the highest richness and diversity of all migrants and that the three hardwood plantation types generally outperformed oil palm in richness and diversity metrics. Despite this, oil palm supported high abundance of several common and widespread species also experiencing range-wide population declines and may therefore play an important role in conserving common species. Mature secondary forest hosted low abundance and diversity of the full migratory community, but high abundance and richness of conservation priority migrants along with native hardwood and teak plantations. Likewise, the percentage of forest cover on the landscape was positively associated with priority migrant abundance and richness but negatively associated with the abundance of migrants in general, highlighting how individual species within the broad group of Neotropical migratory landbirds respond differently to anthropogenic changes in land use. Across all cover types, the retention of tall overstory trees increased the abundance, richness, and diversity of all migrants, which indicates that vertical structural diversity and remnant trees are important habitat features for birds in agricultural landscapes. Our findings show that conservation opportunities exist in hardwood and oil palm plantations, though the species likely to benefit from conservation action will vary among plantation types. For the subset of conservation priority migrants, our results suggest that conservation efforts should combine strategies that retain and restore secondary forest, promote the adoption of native hardwood and teak plantations, and promote the retention of tall, remnant trees in agricultural landscapes.

## Introduction

Nearctic-Neotropical migratory birds are a diverse group facing conservation challenges at multiple life stages. Loss of habitat during any life stage can drive population declines in this guild [1,2], but a disproportionately high number of species that overwinter in tropical broadleaf forests have experienced long-term population declines [3,4]. The expansion of commercial agriculture currently accounts for 70% of the deforestation in Latin America and reduces habitat for many migratory bird species through decreased forest coverage and habitat structural diversity across their overwintering ranges [5,6]. The impact of agricultural expansion and intensification on avifaunal communities has been well-documented within the Neotropics [7], and research consistently shows that a reduction in structural complexity and floristic diversity of a plant community leads to reduced or altered bird community assemblages relative to those occurring within intact forests [7–11].

As agricultural conversion continues to reduce forest area, conservationists have recognized the need to explore opportunities within human-altered, working landscapes [12,13]. Migratory bird conservation planning and research has focused overwhelmingly on a single agricultural system, shade coffee plantations, as an alternative source of habitat [14,15]. Shade coffee plantations that retain structural heterogeneity and native vegetation can provide high quality habitat to overwintering migrants, including the declining guild of tropical broadleaf associates [16–18]. However, the proportion of coffee plantations managed with a diversified shade component has decreased steadily since the 1970s, and over 75% of coffee is now managed with little or no shade [19,20]. In response to the intensification of coffee production, some conservation attention has shifted to land-sparing techniques that promote the retention of forest around full-sun coffee plantations rather than retaining shade trees within plantations [21]. However, the long-term success of a land-sparing approach is uncertain given its dependence on both market forces and stable governance of land zoning across many national and regional jurisdictions [22]. As such, a need exists to investigate conservation opportunities in working landscapes and agroforests beyond the coffee system.

While native forest and shade coffee plantations decrease across tropical landscapes, other types of tree plantations are increasing in coverage [23]. Brazil currently dominates tropical plantation forestry production in the Americas with over 6.5 million ha of exotic pulpwood and hardwood plantations established by 2011, and considerable interest and opportunity exists for these plantation systems to expand within Central America and northern South America [24,25]. Though African oil palm (*Elaeis guineensis*) is a monocot, we considered it within this group of forestry plantation systems given its structural similarities to tree plantations, including trunks that host ferns and other epiphytes, a partially shaded understory, and the presence of leaf litter [26]. Investment in African oil palm has increased dramatically within the Americas, with the area under cultivation doubling in South America and quadrupling in Central America between 2000 and 2016 to total 1.2 million ha of production in the region [27]. Despite the prevalence and expansion of commercial tree and oil palm plantations, relatively few studies have considered their impact on Nearctic-Neotropical migratory bird communities, and we know of no study that has compared migratory bird use among these common plantation types [8,9,28].

To address this knowledge gap, we designed a study to evaluate the potential of hardwood and oil palm plantations to provide habitat to Nearctic-Neotropical migratory birds in the Caribbean lowlands of Guatemala. Specifically, we compared abundance, richness, and diversity of the full migratory bird community and of a subset of forest-associated, conservation-priority species in African oil palm and three types of hardwood plantation: teak (*Tectona grandis*), rubber (*Hevea brasiliensis*), and mixed-native hardwoods. We compared migratory bird use within plantations to a mature secondary broadleaf forest baseline as primary forest no longer exists at low elevations in this region. We furthermore assessed the response of migratory birds to territory and landscape level habitat features in order to identify management strategies that maximize habitat features of conservation importance across the managed cover types.

## Methods

### Study area

**Fig 1:**
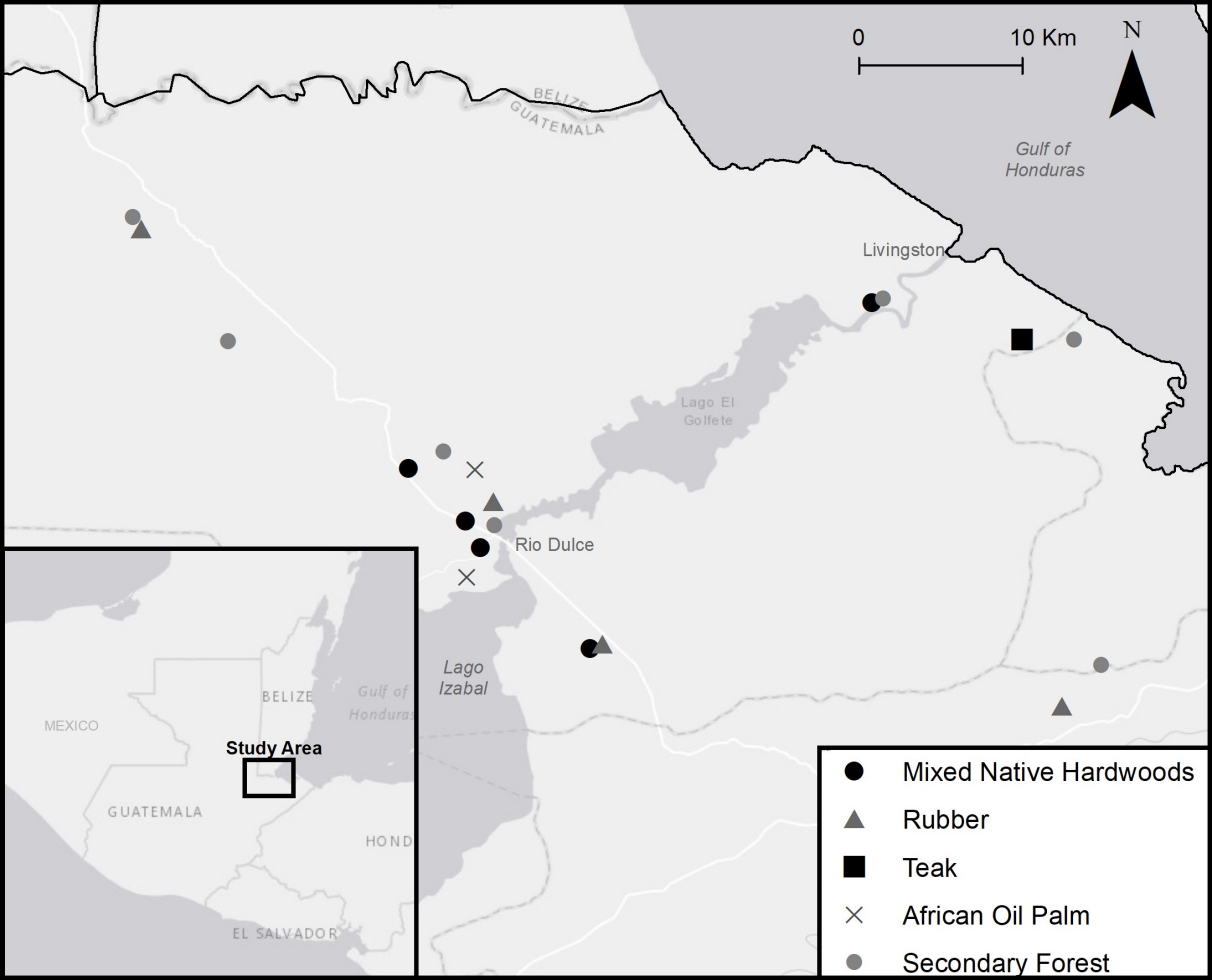
Map of patches surveyed in three types of hardwood plantation (mixed-native hardwoods, rubber, and teak), oil palm plantations, and native secondary forest in the Department of Izabal, Guatemala between 2015 and 2017. Locations of patches of the same cover type within 2 km were averaged to one point.

Between 2015 and 2017, we sampled native secondary forests and four plantation types—mixed-native hardwood plantations, rubber plantations, teak plantations, and African oil palm plantations—in the Caribbean lowlands around Rio Dulce, Department of Izabal, Guatemala (15.66° N, -89.00° W; Fig 1). Secondary forest sites consisted of native overstory tree species with varying degrees of understory development and were < 10 km from the nearest plantation study site. All secondary forest sites had experienced past disturbance due to tree removal but were undisturbed by agricultural production or livestock presence at the time of this study. All study sites were privately owned. The three types of hardwood plantation—teak, rubber, and mixed-native hardwood—were managed for timber or rubber production, and all managed trees were in pole stage, i.e. trees with 10–30 cm diameter-at-breast-height (hereafter DBH). The mixed-native hardwood plantations contained up to 20 native tree species in the overstory with the most common being mahogany (*Swietenia* spp.), white mahogany (*Vochysia guatemalensis*), cocobolo (*Dalbergia retusa*), Monkey-pod (*Samanea saman*), Rosewood (*Dalbergia stevensonii*), and Santa Maria (*Calophyllum brasiliense*), with native cacao (*Theobroma cacao*) occasionally present in the understory. Rubber, teak, and oil palm plantations were managed as monocultures, though understory management varied among sites. We surveyed a range of plantation patch sizes due to high variability in area and configuration of these cover types within the study region (mean patch area ± SE = 116.80 ± 42.71 ha; ranges in S1 Table). Difference in broadleaf tree species cannot be remotely sensed, and no agroforest cover map exists for the region, so our patch size definition was limited to the area of each cover type under contiguous management with a known owner. We accordingly selected and surveyed baseline secondary forests with patch areas within the range of the plantation patch areas surveyed (mean patch area ± SE = 51.6 ± 23.4). Site elevations ranged from 7-m to 358-m with 90% of point-count locations occurring below 100-m elevation. All study sites fell within the tropical wet forest life zone [29]. The landscape comprising the study areas (the extent of the sampled locations plus a 1 km buffer) contained 71% forest, 15% grassland, 9% cultivated land, 3% wetland, and 1% artificial surfaces, according to the GlobeLand30 database [30], but this forest category includes both managed agroforests and native forest.

### Avian surveys

We conducted avian point-count surveys during two consecutive overwintering seasons between 26 January and 2 March 2016 and between 8 November 2016 and 2 February 2017. During the first year, we surveyed points in native secondary forests (*n* = 30), mixed-native hardwood (*n* = 29), rubber (*n* = 30), and teak plantations (*n* = 32) for a total of 121 points. We surveyed 273 points during the second year in native secondary forests (*n* = 60), mixed-native hardwood (*n* = 41), rubber (*n* = 60), teak (*n* = 50) and oil palm plantations (*n* = 62; Fig 1). All points were located 50-m or more from any patch edge and at least 250-m from another point. We recorded all Neotropical migratory birds seen or heard within a 50-m radius during a point-count survey and identified all individuals to species except flycatchers in genus *Empidonax*, which we identified to genus. Counts of individuals of each species were conservative (i.e. if any possibility existed that a recorded individual had moved locations and was being resighted within the same survey period, we counted it as one individual). During the first year, all points were surveyed twice with a 10-min passive survey between dawn and 11:00 AM. A subset of points (*n* = 55; *n*_forest_ = 15, *n*_hardwood_ = 12, *n*_rubber_ = 13, *n*_teak_ = 15) received an additional 11-min survey period directly after the passive surveys in which a 5-min Golden-winged Warbler (*Vermivora chrysoptera*) vocalization and a 5-min owl-mobbing recording (developed by Kenneth Rosenberg and REB, Cornell Lab of Ornithology, 2015) were broadcast at maximum volume on a JAM classic 2.0 portable speaker (UPC 03126207548), followed by a 1-min passive observation period. The owl-mobbing recording contained the typical alarm calls of ten common migratory species plus the songs of Ferruginous Pygmy-Owl (*Glaucidium brasilianum)* and Eastern Screech-Owl (*Megascops asio*). Our preliminary analysis of the first year indicated that the owl-mobbing playback, but not the Golden-winged Warbler playback, significantly increased detection probability for all species. During the second year, we therefore surveyed all points twice with a 10-min passive survey followed by a 5-min owl mobbing playback and a 1-min passive observation period. We did not conduct surveys during periods of high winds or rain. This point-count protocol was approved by the Indiana University of Pennsylvania Institutional Animal Care and Use Committee (IACUC permit number: 11-1617-R2).

In addition to the full migratory bird community, we were interested in species already vulnerable or declining. We therefore selected a subset of five species to form a priority migrant category: Golden-winged Warbler, Blue-winged Warbler (*Vermivora cyanoptera*), Kentucky Warbler (*Geothlypis formosa*), Worm-eating Warbler (*Helmitheros vermivorum*), and Wood Thrush (*Hylocichla mustelina*). These species were selected either because they are birds associated with forest that are experiencing significant population declines (i.e. Wood Thrush, Golden-winged Warbler, Kentucky Warbler) or birds specialized to forage on dead-leaves in the forest midstory (i.e. Blue-winged Warbler and Worm-eating Warbler) which makes them vulnerable to forest loss and degradation of structural habitat [4,31,32]. All five of these species appeared as either Red or Yellow Watch List Species in recent Partners in Flight Landbird Conservation Plans [33,34], signifying they are vulnerable and/or declining and have been identified as a priority for conservation action.

### Habitat surveys

We collected vegetation data at two nested plots with an 11.3-m radius in each 50-m radius point-count area. One plot was located at the site of the point-count survey and the second plot was located 39-m away in a random direction to characterize both the center and the exterior of the 50-m radius point-count area. At each nested plot, we counted the total number of trees (≥ 10 cm DBH) and measured their DBH in an 11.3-m radius. Following Nudds [35], we used a vegetation cover board to measure the density of understory vegetation between 0 and 2-m height at four points in each cardinal direction located 10-m from the nested plot center. We estimated % canopy cover using a spherical densiometer at each of the four vegetation cover board locations. Within the 50-m radius area, we identified the tallest tree and estimated height visually to the nearest 5-m after training to approximate height visually with a clinometer. We also recorded presence or absence of water features within 150-m (i.e. river, stream, or marsh) as the occupancy of Golden-winged Warbler and Blue-winged Warbler (two priority migrants present in in our study region) is associated with the presence of water features at that distance [36].

### Data analysis

We investigated five categories of avian response: overall migrant abundance, overall migrant species richness, overall migrant diversity, priority migrant abundance, and priority migrant species richness. We analyzed avian response categories as a function of 1) detection covariates, 2) Year, 3) cover type, and 4) habitat features. We considered three detection covariates: time of day, understory vegetation density, and use of a playback (used/not used), given evidence time of day and vegetation density affect detection probability [37,38] and our expectation that playback increased detection. Year was a categorical variable (first year or second year) and was included to account for probable temporal variation in abundance. Cover type was a categorical variable containing the four plantation types and secondary forest. We considered six habitat variables that we hypothesized a priori would have relationships with our categories of avian response: basal area, mean understory density (0–2 m), presence of water within 150 m, % canopy cover, % forest cover within 1 km, and height of the tallest tree. Other studies show that vegetation density, canopy coverage, and tree diversity are associated with abundance and richness metrics in coffee agroecosystems [19]. We calculated basal area using the standard formula, Basal Area = 0.00007854×DBH(cm)^2^. As each nested plot comprised ~0.1 acres in area, we summed the basal area of all trees and divided it by 0.0404686 to convert to m^2^/ha and averaged the values of the two nested plots. We averaged % canopy cover and understory density from the eight values calculated in the two nested vegetation plots at each point. Diversity of birds has been shown to relate to trees on a landscape in addition to territory-level habitat characteristics. To account for this, we calculated % tree cover in a 1-km radius with the UMD Forest Cover dataset [39] given evidence birds respond at this distance [40,41]. We started with the % Tree Cover Raster from the year 2000, reclassified all “forest loss” pixels in the 2017 Forest Loss Raster as 0% tree cover, and reclassified all “forest gain” pixels in the 2014 Forest Gains raster as 30% tree cover using the “Raster Calculator” tool in ArcGIS 10.5. Oil palm plantations are occasionally classified as having high % tree cover in this dataset [39], so we masked any areas that were clearly mature oil palm plantations (visible palms with 5–10 m diameter crowns and planted in rows with no retained broadleaf trees) using the Google Earth CNES/Airbus satellite image from 10/8/2017 and classified them as 0% tree cover. We then averaged the % tree cover pixel values in a 1-km radius buffer around each point-count location. We ran one-way analysis of variance (ANOVAs) to calculate differences in these habitat features among each cover type.

#### Models for avian response to cover type and habitat features

All model covariates were additive because we had no a priori reason to believe any interacted, and all continuous variables were standardized prior to inclusion in analyses using the scale function in R to allow for comparisons among coefficients [42]. We determined the Pearson’s correlation among all pairs of continuous variables and did not include variables in the same model if the absolute value was > 0.8. We also checked for multicollinearity among variables and only included variables with a vif < 3 [43].

For the abundance response categories, we fit *N*-mixture models using the ‘unmarked’ package in R [44–46]. These models account for imperfect detection and can include covariates on both the detection and abundance components. We created detection histories for each point-count location in each season (i.e. “0,1,1” for a species with 0 individuals detected on the first survey occasion and with 1 individual detected in the subsequent two survey occasions within a year) and used a Poisson distribution to estimate abundance. The global abundance model included our three detection covariates on the detection side, and Year, Cover Type, and the 6 habitat covariates on the abundance side of the model. We fit all possible subsets of the detection covariates with global abundance models and retained the detection parameters from the best model [47,48]. Time of day and vegetation density were not supported as detection covariates using this method, so we retained only Playback as a detection covariate. We then fit all possible subsets of the abundance covariates. We used the AICcmodavg package to calculate the overdispersion parameter ĉ for the evaluation of both (all migrant and priority migration) global abundance models [49,50]. If ĉ > 1.03, we accounted for the increased variability by using QAIC_*c*_ log likelihood adjustments when comparing models and making predictions.

For the species richness and diversity response categories, we calculated species richness and Shannon’s diversity index of species detected at each point-count location in each season using the vegan package in R [51,52]. We used generalized linear models with a Poisson distribution to estimate overall migrant and priority species richness values of species detected. Similarly, we used a linear model to estimate Shannon’s diversity index for the full complement of migratory species detected at each point. We did not estimate Shannon’s diversity of the priority migrant group, given that it only included five species. For all species richness and diversity models, we included year and use of a playback as detection covariates. For the species richness and diversity response categories, we fit all possible subsets of the following covariates: the six habitat features, a quadratic effect of understory density, year, and use of a playback.

For all models, we selected the subset of competing models using model likelihood cutoff value of ≥ 0.125 and the absence of uninformative parameters [49,53]. Given the presence of multiple competing models, we used model averaged prediction to create all figures displaying the relationships between predictors and avian response metrics.

## Results

We tallied a total of 6,544 detections from 42 Neotropical migratory species and the *Empidonax* genus during our point-counts, and we recorded 1,044 detections of migrants in secondary forest, 1,298 in mixed-native hardwood plantations, 1,528 in rubber plantations, 1,181 in teak plantations, and 1,502 in oil palm plantations. Within the priority migrant category, we detected Golden-winged Warblers (*n* = 21 detections), Blue-winged Warblers (*n* = 37), Kentucky Warblers (*n* = 52), Worm-eating Warblers (*n* = 58), and Wood Thrush (*n* = 263, S2 Table). We detected between 2 and 56 migrants per point (mean = 16.6, SE = 0.4) and 0 to 7 priority migrants per point (mean = 1.1, SE = 0.1). Species richness ranged from one to nineteen migratory species (mean = 7.8, SE = 0.1) and zero to four priority migrant species per point (mean = 0.8, SE = 0.1). Shannon’s diversity index of all migratory species ranged from 1.0 to 15.4 per point (mean = 6.5, SE = 0.0). No habitat variables were correlated. All six of the habitat variables we quantified varied significantly among two or more of the cover types, and averaged values and statistical differences are presented in the Supplementary Material (S1 Fig). Collinearity was not present in any of the model sets. Slight overdispersion was present (ĉ = 1.56) in the overall migrant abundance models so we used QAIC_*c*_ for model selection of this model set and acknowledge overdispersion when making predictions. No species richness or priority migrant models were overdispersed (ĉ = 0.65–1.03). Residuals were homoscedastic for the Shannon’s diversity index models.

**Fig 2:**
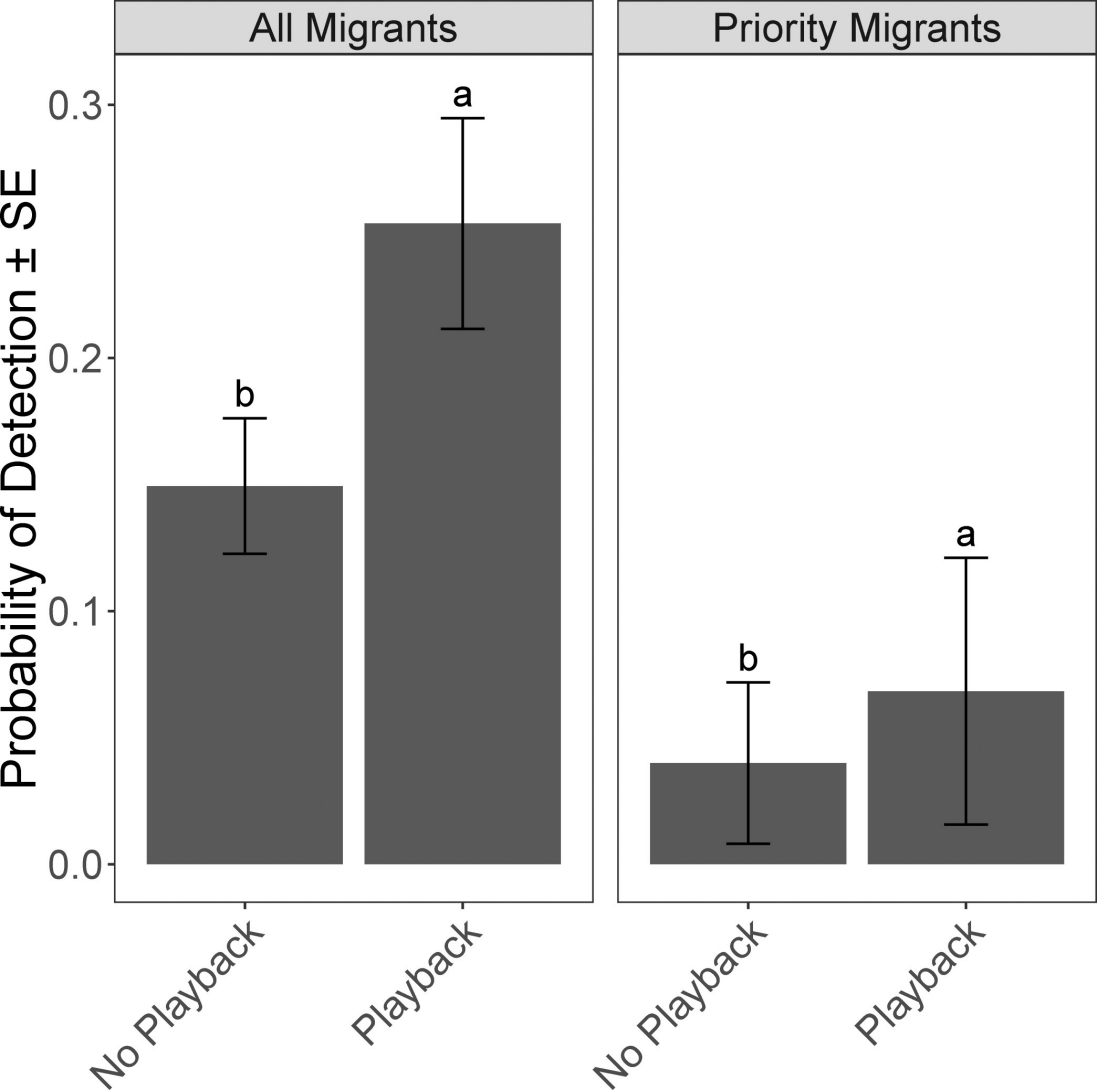
Detection probabilities of all migrants and priority migrants with and without use of an owl-mobbing playback across five cover types in eastern Guatemala during winter 2015–2016 and winter 2016–2017. Created from weighted abundance model averages. Letters indicate significant differences among cover types at p ≤ 0.05.

There were between two and eleven supported models for each model set assessing avian response to habitat characteristics within each of the three cover types (Table 1). Detection probabilities were significantly greater for all migrants and priority migrants with the use of our playback (Fig 2). The abundance of all migrants and the abundance and richness of priority migrants increased significantly in the second year of the study, but the richness and diversity of the “all migrant” category did not vary significantly between years (Tables 1 and 2).

**Table 1:** Supported abundance models and null models for all migrants and priority migrants. Models were selected by likelihood value ≥ 0.125 and the absence of uninformative parameters. Bolded coefficients indicate significance at P ≤ 0.05.

	Detection		Abundance											
Response*	Int	Playback	Int	Year	Cover Type (+/-)**	Height Tallest Tree	% Forest in 1 km	Water in 150m	Und Density	Basal Area	Canopy Cover	df	ΔQAIC_*c*_/ ΔAIC_*c*_	Likelihood
AMA	-1.74	**0.66**	2.78	**0.29**	+	**0.10**	**-0.04**	**0.15**	-	-	-	11	0.00	1.00
AMA	-1.74	**0.66**	2.75	**0.30**	+	**0.10**	-	**0.15**	-	-	-	10	1.00	0.61
AMA null	-1.59	1.15	3.10	-	-	-	-	-	-	-	-	3	222.45	0.00
PMA	-3.20	**0.56**	1.60	**0.96**	+	**0.19**	0.12	-	**-0.17**	-	-	11	0.00	1.00
PMA	-3.18	**0.59**	1.63	**0.95**	+	**0.18**	-	-	**-0.18**	0.07	-	11	0.95	0.62
PMA	-3.12	**0.56**	1.60	**0.97**	+	**0.19**	-	-	**-0.19**	-	-	10	1.49	0.48
PMA	-3.24	**0.58**	1.56	**0.93**	+	**0.19**	**0.12**	-	-	0.07	-	11	3.69	0.16
PMA	-3.19	**0.56**	1.53	**0.94**	+	**0.20**	**0.14**	-	-	-	-	10	3.88	0.14
PMA null	-2.43	1.39	0.81	-	-	-	-	-	-	-	-	3	144.87	0.00

* Response variables defined as All Migrant Abundance (AMA) and Priority Migrant Abundance (PMA).

** “+” indicates the categorical variable “Cover Type” was included in the model and “-” indicates a variable was not included in the model.

**Table 2:** Supported diversity and richness models and null models for all migrants and priority migrants. Models were selected by likelihood value ≥ 0.125 and the absence of uninformative parameters. Bolded coefficients indicate significance at P ≤ 0.05.

Response*	Int	Playback	Year	Cover Type (+/-)**	Height Tallest Tree	% Forest in 1 km	Water in 150m	Und Density	Basal Area	Canopy Cover	df	ΔAIC_*c*_	Likelihood
AMD	2.66	**2.88**	0.46	+	**0.56**	**0.24**	**0.49**	-	-	0.22	12	0.00	1.00
AMD	2.67	**3.24**	-	+	**0.55**	**0.22**	**0.49**	-	-	**0.23**	11	0.38	0.83
AMD	2.82	**2.86**	0.49	+	**0.58**	**0.22**	0.42	-	-	-	11	1.68	0.43
AMD	2.85	**2.86**	0.48	+	**0.58**	**0.23**	-	-	-	0.19	11	2.39	0.30
AMD	2.84	**3.25**	-	+	**0.57**	0.21	0.43	-	-	-	10	2.41	0.30
AMD	2.79	**3.20**	-	+	**0.56**	-	**0.49**	-	-	0.21	10	2.45	0.29
AMD	2.86	**3.24**	-	+	**0.56**	**0.22**	-	-	-	0.19	10	2.92	0.23
AMD	2.97	**2.85**	0.50	+	**0.59**	**0.22**	-	-	-	-	10	3.05	0.22
AMD	2.93	**2.85**	0.45	+	**0.59**	-	0.42	-	-	-	10	3.70	0.16
AMD	2.99	**3.24**	-	+	**0.58**	0.21	-	-	-	-	9	3.87	0.14
AMD	2.94	**3.20**	-	+	**0.58**	-	0.43	-	-	-	9	3.96	0.14
AMD null	6.53	-	-	-	-	-	-	-	-	-	2	181.89	0.00
AMR	1.28	**0.64**	-	+	**0.09**	-	0.06	-	-	-	8	0.00	1.00
AMR	1.31	**0.64**	-	+	**0.09**	-	-	-	-	-	7	0.07	0.96
AMR null	2.05	-	-	-	-	-	-	-	-	-	1	174.84	0.00
PMR	-1.07	**0.88**	**0.47**	+	**0.18**	-	-	**-0.21**	-	-	9	0.00	1.00
PMR	-1.21	**0.87**	**0.45**	+	**0.19**	0.11	-	-	-	-	9	4.02	0.13
PMR null	-0.20	-	-	-	-	-	-	-	-	-	1	108.80	0.00

* Response variables defined as All Migrant Diversity (AMD), All Migrant Species Richness (AMR), and Priority Migrant Species Richness (PMR).

** “+” indicates the categorical variable “Cover Type” was included in the model and “-” indicates a variable was not included in the model.

### Cover types

**Fig 3:**
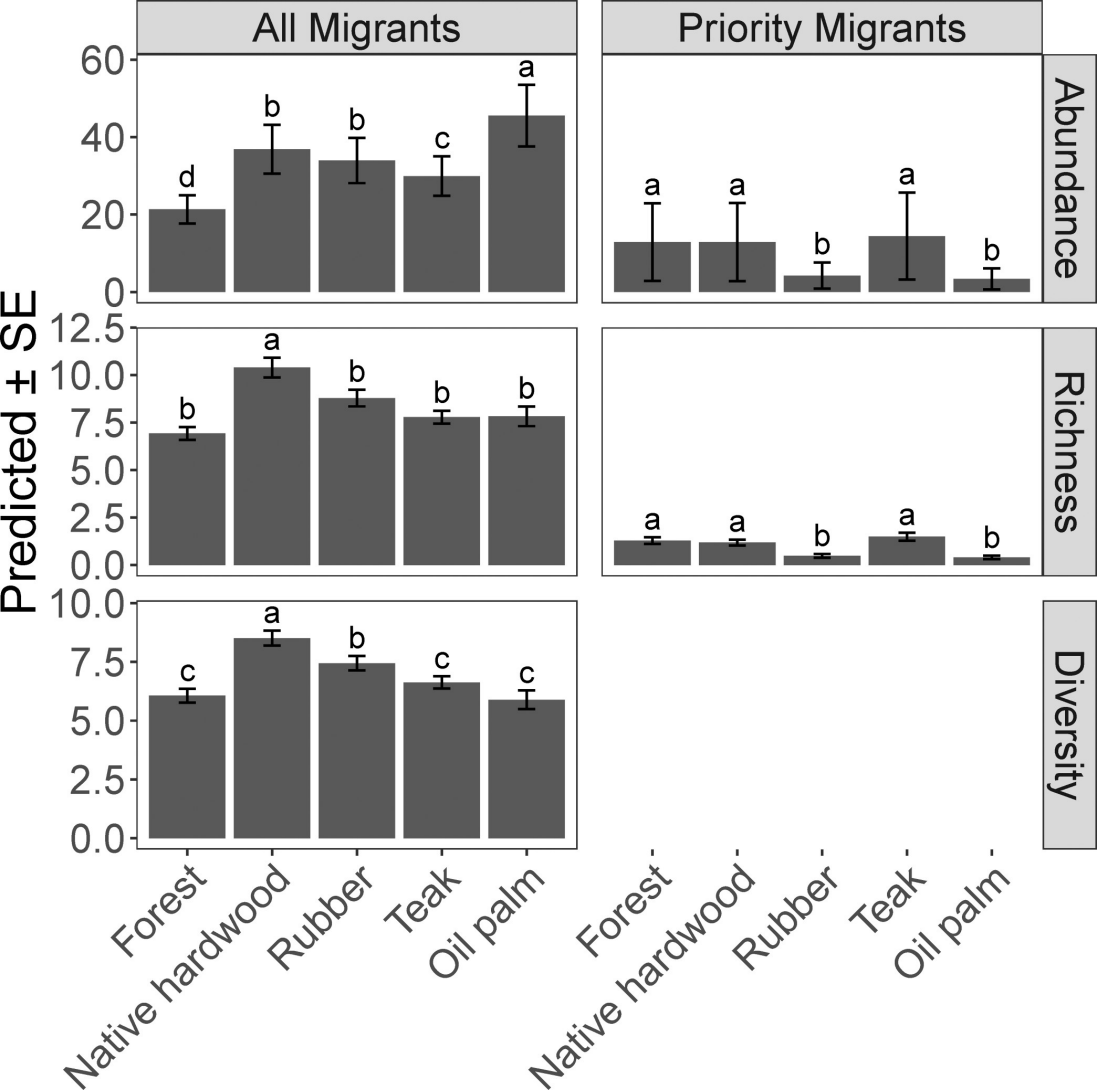
Model averaged predictions for five avian response categories to five cover types surveyed in eastern Guatemala during winter 2015–2016 and winter 2016–2017. Letters indicate significant differences among cover types at p ≤ 0.05.

Cover type was a supported covariate in all competing models for all metrics of migratory bird abundance, richness, and diversity (Tables 1 and 2). In the “all migrant” category, cover type interacted differently with abundance than richness and diversity metrics, while cover type showed the same relationships with abundance and richness for the “priority migrant” category (Fig 3). Secondary forest had low abundance, richness, and diversity of all migrants, but high abundance and richness of priority migrants (Fig 3). Of all the cover types, mixed native hardwoods had the greatest richness and diversity of all migrants and similarly showed high abundance and richness of priority migrants (Fig 3). The other two hardwood cover types, rubber and teak, showed opposite relationships between the “all migrant” and “priority migrant” categories; rubber supported greater abundance and diversity of all migrants while teak supported greater abundance and richness of priority migrants (Fig 3). Finally, oil palm supported the greatest overall abundance of migratory birds but had low richness and diversity of all migrants and low abundance and richness of priority migrants (Fig 3).

### Habitat features

**Fig 4:**
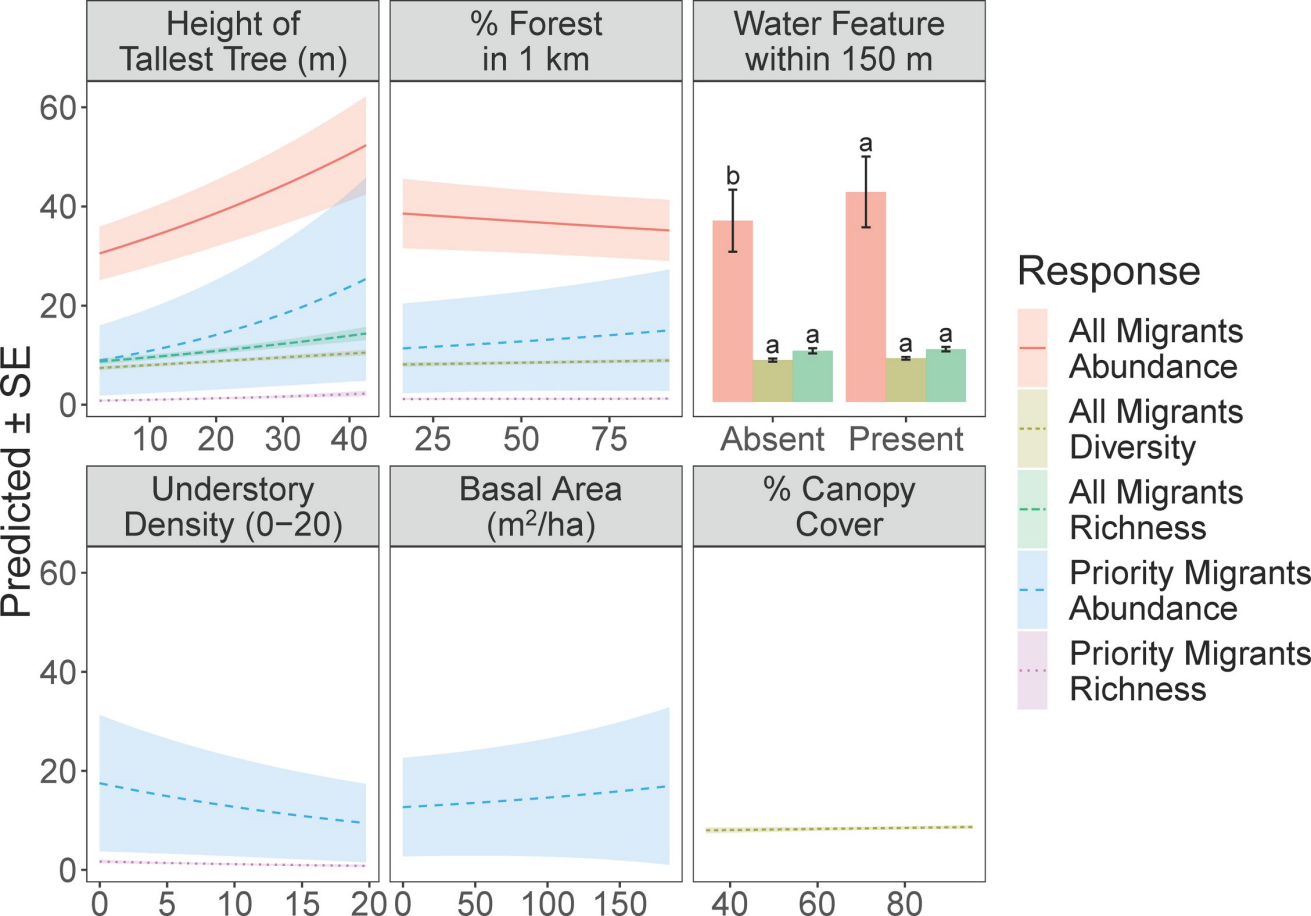
Model averaged predictions for five avian response categories to six habitat and landscape level variables across 5 cover types in eastern Guatemala during winter 2015–2016 and winter 2016–2017. Letters indicate significant differences among cover types at p ≤ 0.05.

Height of the tallest tree was the only habitat variable that appeared in all supported models, and it predicted significant increases in all abundance, richness, and diversity metrics (Tables 1 and 2, Fig 4). Percent forest in a 1 km radius predicted a significant increase in all migrant diversity and slight increases in the abundance and richness of priority migrants, while the overall abundance of migrants decreased with percent forest on the landscape (Fig 4). The abundance of all migrants increased significantly with presence of a water feature, while richness and diversity increased insignificantly, and priority migrants showed no response to the variable (Fig 4). Migrant diversity increased slightly with canopy cover, and the abundance of priority migrants increased slightly with basal area (Fig 4). Finally, both the abundance and richness of priority migrants decreased significantly with the density of the understory between 0 and 2 m (Fig 4).

## Discussion

Our results showed that in the Caribbean lowlands of Guatemala, native and exotic hardwood plantations, oil palm plantations, and secondary forest all provide habitat to Neotropical migrants. Abundance, richness, and diversity of migrants vary significantly among cover types, with mixed native hardwoods supported the greatest overall richness and diversity of migrants, while oil palm hosted the highest abundances, and secondary forest supported high conservation priority migrants more than the full migratory community. While some relationships between cover type and habitat associations were different for conservation-priority species than for the migratory bird community in general, we showed that two features—tall trees and water features—provide a benefit to the entire migratory community. These results allow us to critically assess the conservation potential of hardwood and oil palm plantations within ecoregions similar to our study area and recommend specific management actions to improve the value of agroforestry plantations for Neotropical migratory birds.

Height of the tallest tree within a 50-m radius was consistently the most significant predictor of abundance, richness, and diversity of migratory birds in all surveyed cover types. Given that each plantation had relatively uniformly sized trees or palms, height of the tallest tree serves as a metric of the vertical, structural diversity of a plot. Our result agrees with previous studies correlating high structural diversity of habitat with high diversity of avian assemblages in tropical systems [8,16,54]. Beyond vertical structure, remnant forest trees typically host more vines, epiphytes, and other microhabitat features than planted trees, thereby enhancing the multiple metrics of biodiversity in agroecosystems (reviewed in [55]). In our study region, scattered remnant trees were often retained within the cover types we surveyed, especially along riparian corridors and steep slopes, and were often the tallest trees within a plot. The strong positive relationship between height of the tallest tree and our metrics of abundance, richness, and diversity strongly suggests that the retention of large trees increases the habitat value of a plantation for migratory birds. Conservation planning should therefore incorporate specific recommendations to retain vertical diversity and remnant trees wherever possible in hardwood and oil palm plantations.

Presence of a water feature, such as a stream or river, predicted increases in the abundance of the overall migratory community. Riparian corridors and areas around small streams in tropical forests are known to support significantly higher arthropod abundance than the surrounding landscape, thereby providing important food resources for insectivorous birds [56]. Our result agrees with research done in Mexico that documented significant increases in the abundance of migratory birds, but not resident birds, around riparian corridors in human-disturbed landscapes [57]. Interestingly, our point-counts in oil palm plantations were more likely to be close to a water feature than point-counts in other cover types, and oil palm plantations accordingly hosted greater abundance of migratory birds than other cover types. The lack of a response from conservation-priority migrants to water features was unexpected, especially given evidence in other ecosystems (mid-elevation humid forest and coffee farms) that Golden-winged Warbler and Blue-winged Warbler do respond positively to riparian corridors [36]. However, in our study region, tall trees, understory density, basal area, and amount of forest on the landscape were more important explainers of the abundance and richness of conservation-priority species than simply presence of a water feature. This highlights how habitat relationships can change in different ecosystems or regions. Despite this, protection of the vegetation around streams should remain a priority for the general conservation of Neotropical migratory communities given their importance to the overall migratory community and given the well-established importance of riparian vegetation for maintaining water quality and hydrological processes [58].

Cover type was a supported variable in all competing models, indicating that abundance, richness, and diversity metrics were explained by plantation type in addition to the habitat features discussed above. The three hardwood plantation types generally outperformed oil palm in avian species richness and diversity metrics. Mixed-native hardwoods and rubber supported the greatest combined abundance, species richness, and diversity of the full migratory community, while teak supported a less diverse community. However, mixed-native hardwoods and teak significantly outperformed rubber plantations with respect to the abundance and richness of conservation-priority migrants. The difference in priority migrant use of teak and rubber plantations is striking, given that both are exotic monocultures with similarly sized trees. These two plantation systems varied significantly in understory management, with no active understory management and high vegetation density (0–2 m) in the teak plantations versus active understory removal and very low vegetation density in the rubber plantations. Furthermore, teak leaves are larger than rubber and seasonally deciduous, which may affect the associated arthropod community that the priority migrants would target for food. As many of the priority migrants forage preferentially on arthropods in leaf litter suspended in the midstory or understory [31,59], it is likely that the well-developed understory in the teak plantation provided this type of habitat feature while the heavily managed understory of rubber plantations did not. Both mixed-native hardwoods and teak plantations supported abundances and richness of priority migrants similar to those of secondary forest. As such, any management actions focused on increasing the abundance of migrants within teak or mixed-native hardwood plantations are likely to benefit Neotropical migrants of conservation concern. This result is especially important given that teak monocultures are a common and expanding hardwood plantation system and an important source of income in Mesoamerica (Mexico, Central America, and the Caribbean; [60]).

Our demonstration that mixed-native hardwood plantations support conservation-priority migrants as well as a diverse overall migratory community highlights an exciting opportunity to integrate Neotropical migratory bird conservation efforts with native hardwood plantation forestry. Increasingly, foresters and landowners are demonstrating interest in developing plantations that incorporate native hardwoods [61]. Mesoamerica has high regional diversity of native trees with timber value [62,63], and while mixed-native hardwood plantations are still relatively rare on the landscape, studies from Costa Rica showed that these plantations are economically viable both to investors and to landowners when managed on a 25-year rotation [64,65]. Under optimal conditions in Costa Rica, mixed-native hardwood plantations accrued greater net profit compared to exotic monoculture plantations [65]. Native tree plantations have long been recommended as a means to conserve biodiversity on working lands, with an emphasis on cultivating multiple species of native trees together whenever possible [66,67]. Mixed-native hardwood plantations also disperse native seeds, support a diverse native understory, provide habitat to native bird and bat species, and aid in surrounding reforestation efforts [68]. Despite the conservation potential of mixed-native hardwood plantations, attempts to promote their adoption in tropical developing countries have been criticized for poor design, specifically with regards to market analysis, and insufficient extension services to investors and landowners [63]. Increasing the adoption of mixed-native hardwood plantations will require investment to develop plantation management regimes and technical knowledge that can be accessed readily by the public, local governments, and nongovernmental agencies[69]. Our results suggest that this type of investment will have a high conservation value for migratory birds in addition to potential income generation.

Oil palm plantations supported the highest abundance of migratory birds of all cover types, showing that this intensive and expansive monoculture system does provide habitat for migratory birds. However, oil palm also supported the lowest combined richness and diversity of migratory species, and low abundance and richness of conservation-priority migrants, indicating that it has less potential than the hardwood plantation types to host a diverse community of migratory birds. Though our results and other studies indicate that forest-associated species are largely excluded by oil palm [70], conservation opportunities do exist in this cover type. Two of the most common species that we detected in oil palm, Yellow Warbler (*Setophaga petechia*) and Common Yellowthroat (*Geothlypis trichas*), are experiencing rangewide population declines [4], and oil palm plantations may play a role in helping to keep these common birds common. Future work should explore habitat quality within oil palm plantations for these common, but declining species to determine what specific management actions will allow oil palm to provide the highest quality habitat for the migratory species that do select this plantation type.

Caution is needed when assessing the conservation potential of disturbed, human-dominated landscapes with use and abundance data. Most studies of overwintering migratory birds have shown that densities correlate with food availability and that individual birds will track temporal shifts in food by moving to areas with more resources [14,71,72]. Indeed, density of overwintering migrants has been shown to predict overwintering survival and body condition metrics in both in natural and agricultural habitats in Mesoamerica [73]. As such, we expect that our results reflect real differences in resource availability in this overwintering landscape. However, human-disturbed landscapes may have impacts on migratory songbird survival that are not linked to food availability, such as an increase in predator abundance or diversity, which could increase stress-levels, costly predator-avoidance behaviors, or rates of depredation [74,75]. Indeed, Villaseñor [76] described significantly higher stress levels for migratory birds using disturbed and fragmented riparian habitats in Mexico compared with intact riparian forests, although the physical condition and abundance of individuals were similar to those of the conspecifics in less disturbed habitats. Furthermore, while coffee landscapes have been shown to provide good quality habitat to overwintering migrants, as measured by consistent gains in weight over the winter season [17], this work remains to be done for hardwood and oil palm plantations. Metrics of survival, body condition, and stress levels were beyond the scope of this study, but future work on the physiology and demographics of this overwintering migratory bird community would help add information about habitat quality to our habitat use results.

In our assessment of migratory bird use metrics in mature secondary forest, we found that native forest patches supported high abundance and richness of our subset of conservation-priority migrants but supported low abundance and richness of the full migratory community. Likewise, the percentage of forest cover on the landscape was positively associated with priority migrant abundance and richness but negatively associated with the abundance of migrants in general. This difference is striking and provides insight into how individual species within a broad group like Neotropical migratory landbirds may respond differently to anthropogenic land use changes based on their ability to use human-disturbed landscapes. Forest coverage in Mesoamerica has declined steadily since 1970 [77], correlating with the long-term population declines in the forest-associated priority migrants. Guatemala alone has lost over 1 million hectares of forest (~10% of the total forested area) since 2001, and 4.6 million hectares have been lost across the Mesoamerican region in that time [68]. While knowledge is incomplete about the direct effect of nonbreeding habitat loss on the population trends of these species, forest loss is assumed to contribute to and possibly drive these population declines [2,36]. Our finding that forest-associated, conservation-priority species will use hardwood plantations, especially when the habitat features discussed previously are retained, shows that conservation of these priority species can occur jointly with income-generating activities in tropical working landscapes. Conservation efforts for this group should therefore seek to combine strategies that retain and restore secondary forest, promote native hardwoods and teak plantations, and promote the retention of tall, remnant trees in agroforestry systems to help ensure forest-associated migrants remain a prominent feature of the migratory bird community.

## Supporting information

S1 Fig Habitat characteristics of plantations and secondary forest surveyed in Guatemala, 2016. Letters denote significance at p ≤ 0.05 in one-way ANOVAs.
(TIF)

S1 Table Size of secondary forest and plantation patches surveyed in the Department of Izabal, Guatemala during winter 2015–2016 and winter 2016–2017. (DOCX)

S2 Table Total detections of conservation-priority migrants across all avian point count surveys in Guatemala during winter 2015–2016 and winter 2016–2017. (DOCX)
