# Supplementary figures and images for: Conservation of Neotropical migratory birds in tropical hardwood and oil palm plantations
Source: PLoS One. 2018 Dec 31;13(12):e0210293. doi: 10.1371/journal.pone.0210293 (PMC6312276; doi:10.1371/journal.pone.0210293)

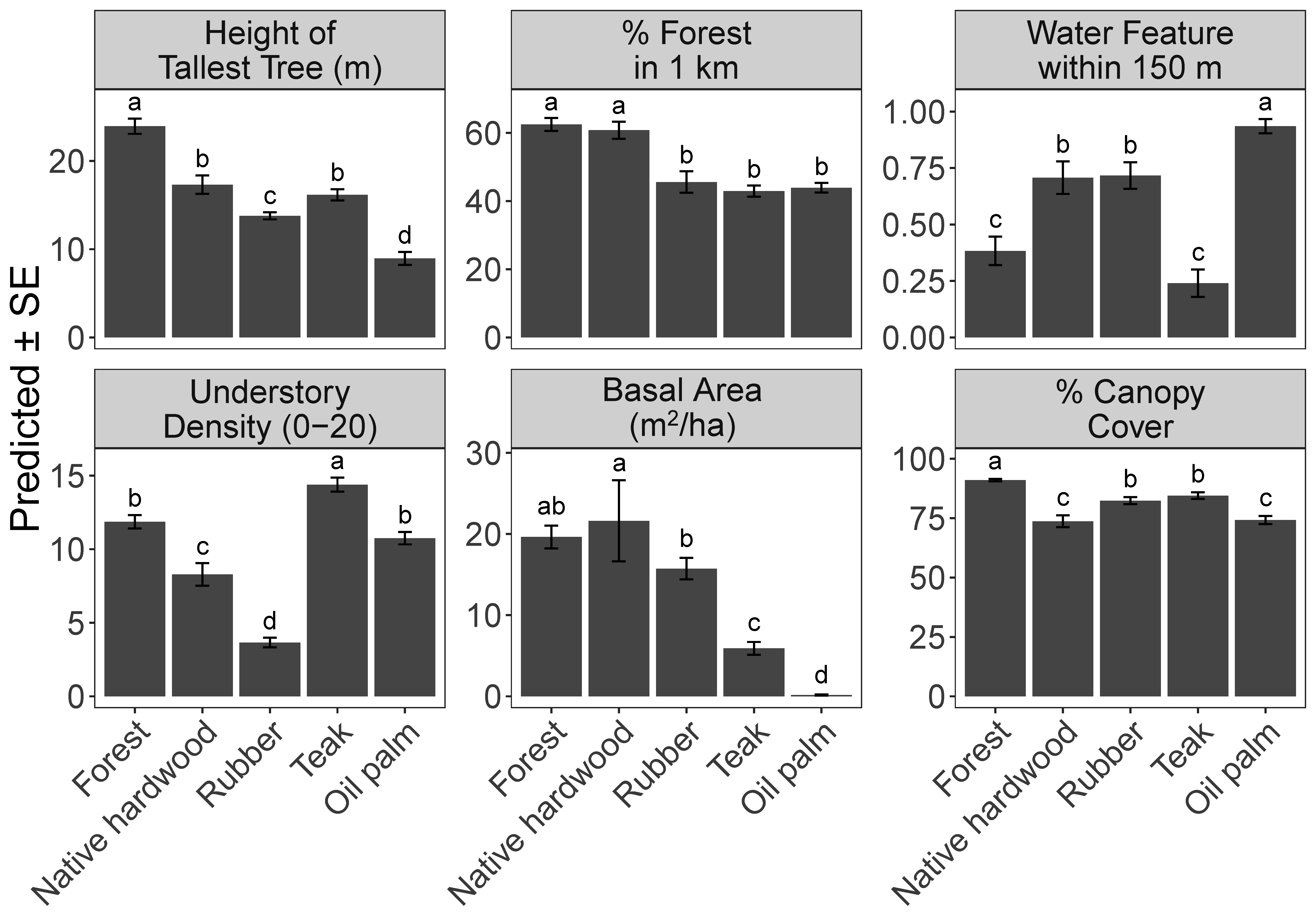

Supplement: S1 Fig — Letters denote significance at p ≤ 0.05 in one-way ANOVAs. (TIF) [file pone.0210293.s001.tif]
