# Supplementary material for: Conservation of Neotropical migratory birds in tropical hardwood and oil palm plantations
Source: PLoS One. 2018 Dec 31;13(12):e0210293. doi: 10.1371/journal.pone.0210293 (PMC6312276; doi:10.1371/journal.pone.0210293)
Supplement: S1 Table — (DOCX) [file pone.0210293.s002.docx]

| Cover type | Number of sites | Site size (ha, mean ± SE) | Range of site size (ha) |
| --- | --- | --- | --- |
| Secondary Forest | 8 | 51.6 ± 23.4 | 1.8 – 204.6 |
| Mixed-native Hardwood | 4 | 40.5 ± 20.2 | 2.2 – 90.3 |
| Rubber | 4 | 118.5 ± 59.8 | 12.7 – 289.9 |
| Teak | 1 | 585.3 | 585.3 |
| Oil Palm | 3 | 137.8 ± 46.4 | 68.6 – 226.0 |
