# Supplementary material for: Conservation of Neotropical migratory birds in tropical hardwood and oil palm plantations
Source: PLoS One. 2018 Dec 31;13(12):e0210293. doi: 10.1371/journal.pone.0210293 (PMC6312276; doi:10.1371/journal.pone.0210293)
Supplement: S2 Table — (DOCX) [file pone.0210293.s003.docx]

| Cover type | Blue-winged Warbler | Golden-winged Warbler | Kentucky Warbler | Wood Thrush | Worm-eating Warbler | Total |
| --- | --- | --- | --- | --- | --- | --- |
| Forest | 8 | 2 | 27 | 94 | 22 | 153 |
| Mixed-Native Hardwood | 17 | 15 | 9 | 61 | 6 | 108 |
|  |  |  |  |  |  |  |
| Rubber | 10 | 3 | 3 | 27 | 3 | 46 |
| Teak | 1 | 1 | 13 | 61 | 26 | 102 |
| Oil Palm | 1 | 0 | 0 | 20 | 1 | 22 |
